# Draft Genome Sequence of Pseudoalteromonas luteoviolacea HI1, Determined Using Roche 454 and PacBio Single-Molecule Real-Time Hybrid Sequencing

**DOI:** 10.1128/genomeA.01590-14

**Published:** 2015-02-19

**Authors:** Audrey Y. Asahina, Michael G. Hadfield

**Affiliations:** Pacific Biosciences Research Center, Kewalo Marine Laboratory, University of Hawaii at Manoa, Honolulu, Hawaii, USA

## Abstract

We report here the 6.0-Mb draft genome assembly of Pseudoalteromonas luteoviolacea strain HI1 using Roche 454 and PacBio single-molecule real-time hybrid-sequencing analysis. This strain is of biological importance since it has the capacity to induce the settlement and metamorphosis of the serpulid polychaete Hydroides elegans and the coral *Pocillopora damicornis*.

## GENOME ANNOUNCEMENT

Pseudoalteromonas luteoviolacea strain HI1 was isolated from biofilm in a seawater table at the Kewalo Marine Laboratory (Honolulu, HI, USA) (1) and identified from 16S rDNA sequencing. Currently, there are 2 draft genomes published for P. luteoviolacea in NCBI: P. luteoviolacea 2ta16 and P. luteoviolacea ATCC 29581 (2). However, P. luteoviolacea ATCC 29581 appears to have been misclassified based on a 16S rRNA phylogenetic analysis and is recommended to be reclassified as a distinct species more closely related to Pseudoalteromonas ulvae (2).

*Pseudoalteromonas* spp. have been found to produce a variety of compounds that possess a range of effects that include antibacterial (3–5), antifouling (6), and algicidal (7) activities. P. luteoviolacea HI1, particularly, has been studied for its effects on the settlement and metamorphosis of the biofouling serpulid polychaete Hydroides elegans (1, 8, 9) and the coral *Pocillopora damicornis* (10). Studies have identified the region of this bacterial genome associated with settlement of H. elegans (8). Most recently, a functional component within this set of genes that is associated with the settlement and metamorphosis of H. elegans has been reported to be a large complex of phage tail-like elements (9).

Genomic DNA was submitted to New Mexico State University for Roche 454 sequencing, which resulted in 198,444 reads with an average mean read length of 418.56 bp, totaling 83,056,281 nucleotides. Assembly of the Roche 454 reads with Sequencher generated 172 contigs. The Roche 454 contigs were then supplemented by aligning the contigs with the PacBio single-molecule real-time (SMRT) long reads. For PacBio SMRT sequencing, genomic DNA was submitted to the National Center for Genome Resources. A single library on 1 SMRT cell was prepared, resulting in 82,296 raw reads with a mean read length of 5,344 bp, totaling 439,749,929 nucleotides. Generated reads were then introduced into the Hierarchical Genome Assembly Process (HGAP), assembled with the Celera Assembler, and polished with Quiver. To create the hybrid assembly, the Roche 454 and HGAP assemblies were combined using Minimus (11) and rescaffolded using PacBio’s hybrid assembler program, resulting in 10 scaffolds containing 4 gaps within 2 scaffolds. The 6.0-Mb genome had a total GC content of 42% with 172 RNAs (44 rRNAs and 128 tRNAs). Annotation was performed with the Prokaryotic Genome Annotation Pipeline (PGAP), Rapid Annotations using Subsystem Technology (RAST) server (12, 13), and manually curated with GenePrimp (14). RAST predicted 5,326 coding sequences, 14 of which were identified as phage/prophage components. One of these phage components has been identified as the phage tail-fiber protein, which has been reported to be involved in host-cell receptor binding (15). RAST also identified type I, II, IV, VI, and VIII secretion-system components. Secondary metabolites were identified using antiSMASH (16), and 3 CRISPR regions were recognized using the CRISPERfinder program (http://crispr.u-psud.fr/Server). The draft genome of *P. luteoviolacea* HI1 will assist in uncovering secondary metabolites, enzymes, and other compounds that may be of biological and biotechnological importance and which may be useful in elucidating the mechanisms involved in the settlement and metamorphosis of *H. elegans* and other marine invertebrates.

### Nucleotide sequence accession numbers.

This whole-genome shotgun project has been deposited at DDBJ/EMBL/GenBank under the accession number JWIC00000000. The version described in this paper is version JWIC01000000.
